# Amino acid derivatives are substrates or non-transported inhibitors of the amino acid transporter PAT2 (slc36a2)

**DOI:** 10.1016/j.bbamem.2010.07.032

**Published:** 2011-01

**Authors:** Noel Edwards, Catriona M.H. Anderson, Kelly M. Gatfield, Mark P. Jevons, Vadivel Ganapathy, David T. Thwaites

**Affiliations:** a Epithelial Research Group, Institute for Cell & Molecular Biosciences, Faculty of Medical Sciences, Newcastle University, Framlington Place, Newcastle upon Tyne, NE2 4HH, UK; b Department of Biochemistry and Molecular Biology, Medical College of Georgia, Augusta, GA 30912, USA

**Keywords:** 1-ACHC, 1-aminocyclohexanecarboxylic acid, C2-ACHC, cis-2-aminocyclohexanecarboxylic acid, C4-ACHC, cis-4-aminocyclohexanecarboxylic acid, CHDP, cis-4-hydroxy-d-proline, CHLP, cis-4-hydroxy-l-proline, DHP, 3,4-dehydro-d,l-proline, Guv, guvacine, 3-IPA, indole-3-propionic acid, Isoguv, isoguvacine, Isonip, isonipecotic acid, Me-Trp, α-methyl-d,l-tryptophan, 1-Me-d-Trp, 1-methyl-d-tryptophan, 1-Me-l-Trp, 1-methyl-l-tryptophan, Nip, nipecotic acid, OH-Trp, 5-hydroxy-l-tryptophan, d-Pip, d-pipecolic acid, l-Pip, l-pipecolic acid, Sar, sarcosine, SLC, solute carrier family, T2-ACHC, trans-2-aminocyclohexanecarboxylic acid, TCA, l-thiaproline (l-thiazolidine-4-carboxylic acid), T4HP, trans-4-hydroxy-l-proline, T3HP, trans-3-hydroxy-l-proline, Trypt, tryptamine, PAT2, Amino acid transport, SLC36, PAT1, Tryptophan, Inhibitor

## Abstract

The H^+^-coupled amino acid transporter PAT2 (SLC36A2) transports the amino acids proline, glycine, alanine and hydroxyproline. A physiological role played by PAT2 in amino acid reabsorption in the renal proximal tubule is demonstrated by mutations in *SLC36A2* that lead to an iminoglycinuric phenotype (imino acid and glycine uria) in humans. A number of proline, GABA and tryptophan derivatives were examined to determine if they function either as transported substrates or non-transported inhibitors of PAT2. The compounds were investigated following heterologous expression of rat PAT2 in *Xenopus laevis* oocytes. PAT2 function was characterised by: radiotracer uptake and competition (cis-inhibition) studies; radiotracer efflux and trans-stimulation; and measurement of substrate-induced positive inward current by two-electrode voltage-clamp. In general, the proline derivatives appeared to be transported substrates and the relative ability to induce current flow was closely related to the inhibitory effects on PAT2-mediated l-[^3^H]proline uptake. In contrast, certain heterocyclic GABA derivatives (e.g. l-pipecolic acid) were translocated only slowly. Finally, the tryptophan derivatives inhibited PAT2 function but did not undergo transport. l-Proline uptake was inhibited by 5-hydroxy-l-tryptophan (IC_50_ 1.6 ± 0.4 mM), α-methyl-d,l-tryptophan (3.5 ± 1.5 mM), l-tryptophan, 1-methyl-l-tryptophan and indole-3-propionic acid. Although neither 5-hydroxy-l-tryptophan nor α-methyl-d,l-tryptophan were able to elicit inward current in PAT2-expressing oocytes both reduced the current evoked by l-proline. 5-Hydroxy-l-tryptophan and α-methyl-d,l-tryptophan were unable to trans-stimulate l-proline efflux from PAT2-expressing oocytes, confirming that the two compounds act as non-transported blockers of PAT2. These two tryptophan derivatives should prove valuable experimental tools in future investigations of the physiological roles of PAT2.

## Research Highlights

►PAT2 (SLC36A2) transports proline, glycine, alanine and hydroxyproline. ►Heterocyclic proline derivatives are transported substrates. ►Heterocyclic GABA derivatives are translocated slowly. ►Tryptophan derivatives (e.g. α-methyl-d,l-tryptophan) are non-transported inhibitors.

## 1 Introduction

The SLC36 (solute carrier 36) family of transporter-related genes consists of four members (*SLC36A1–4*) in all mammalian genomes investigated thus far [1–3]. The SLC36 transporters, along with the SLC32 and SLC38 families (categorised using the nomenclature of the Human Genome Organisation [4]), form the β-group cluster of amino acid transporters [5] and are members of the Amino Acid/Auxin Permease (AAAP) family. The AAAP family is contained within the amino acid/polyamine/organocation (APC) transporter superfamily, as classified using the nomenclature of the Transport Classification Database [6].

The *SLC36A1* gene encodes a H^+^-coupled amino acid transporter named variously as LYAAT1 or PAT1 [7]. cDNAs for PAT1 have been isolated from rat [8], mouse [9], human [10] and rabbit [11]. When expressed in a heterologous system, PAT1 produces an amino acid transporter that has the functional characteristics of a transport system (named system PAT for Proton-coupled Amino acid Transporter) previously identified at the apical membrane of monolayers of the human intestinal epithelial cell line Caco-2 [12,13]. PAT1 has been identified as the molecular correlate of the imino acid carrier [14], a transport system identified functionally in rat small intestine as long ago as the 1960s [15–17]. The potential importance of PAT1 in amino acid absorption in the mammalian small intestine is demonstrated by immunolocalisation of PAT1 protein to the luminal surface of Caco-2 cell monolayers, and both human and rat small intestine [7,14]. The substrate specificity of PAT1 has been explored in great detail and PAT1 transports a wide variety of l- and d-amino and imino acids in α-, β- and γ-orientations, and a large number of heterocyclic compounds and orally-delivered drugs related to proline and GABA (for examples see [7,9,13,14,18–23]). Recently PAT1 has been shown to transport the conditionally-essential amino acid taurine [24] and the photosensitising anti-cancer agent δ-aminolevulinic acid [25].

In contrast to PAT1, much less is known about the other members of the SLC36 family. SLC36A3/PAT3 and SLC36A4/PAT4 remain orphan transporters with no known function. Based upon homology to PAT1 (for example, human PAT1 and PAT2 (SLC36A2) share 72% identity in amino acid sequence), PAT2 was isolated from mouse [9], rat [1] and human [2]. Like PAT1, PAT2 functions as an H^+^-coupled amino acid transport system when expressed in *Xenopus laevis* oocytes or human RPE cells. PAT2 has a higher affinity for its substrates, when compared to PAT1, but transports a narrower range of compounds [9,26,27]. Despite the substrate specificity of this transport system being characterised in some detail, the physiological role(s) of the transporter is uncertain as, before isolation of the cDNAs, an endogenous transport system with obvious PAT2-like characteristics had not previously been identified in any tissue. However, a few clues to the likely physiological functions of PAT2 in neuronal and renal tissues have emerged over recent years. Immunolocalisation of PAT2 to the endoplasmic reticulum, recycling endosomes and plasma-membrane of neurones in mouse brain [28] suggests that PAT2 may be involved in amino acid movement in neuronal tissues. A Na^+^-independent, relatively low affinity, transporter of glycine, alanine and proline had previously been identified in rat CNS tissues that demonstrates some similarity in function to PAT2 [29,30]. In addition, PAT2 protein (named Tramdorin 1 in the study) was immunolocalised to myelinating Schwann cells suggesting a role in amino acid supply during differentiation [31]. However, the strongest evidence for a physiological role of PAT2 comes from investigations by Bröer and colleagues [32,33]. The *SLC36A2* (*PAT2*) gene was identified as the major gene responsible for iminoglycinuria, a renal defect characterised by reduced reabsorption of glycine, proline and hydroxyproline (Online Mendelian Inheritance in Man, OMIM: 242600). In five pedigrees a single amino acid substitution (G87V) in PAT2 was identified [32]. Expression of the G87V PAT2 mutant in *Xenopus* oocytes produced a transporter with reduced activity compared to wild-type PAT2 because of decreased affinity for proline and glycine [32]. In a separate pedigree, a splice donor site mutation in the first intron of *SLC36A2* was identified. This mutation produced a truncated protein with no function [32]. Thus, a physiological role of PAT2 in the renal proximal tubule is in the reabsorption of glycine, proline and hydroxyproline. This role is emphasised by the recent finding that reduced PAT2 expression in the apical membrane of mouse proximal tubule is responsible in part for the developmental iminoglycinuria observed in neonatal animals [33].

Although major advances have been made over recent years in our understanding of membrane transporter function in mammalian tissues it is also likely that many unidentified transport systems will play unique physiological roles. These roles can be difficult to define because the function of an individual transporter may be masked, for example, due to coexpression with other transport systems, low level of expression, or limited expression in a sub-population of cells within a mixed tissue. In the case of amino acid transporters, a major difficulty in attributing transport of any individual compound to any single transport system is due to the overlapping substrate specificities and affinities observed between many transporters. The development of a panel of selective substrates or, preferably, non-transported inhibitors will allow discrimination between movements through distinct transport systems. Thus, the objective of this investigation was to identify non-transported inhibitors of the H^+^-coupled amino acid transporter PAT2. A previous study [27] emphasised the potential of heterocyclic compounds as lead candidates for this investigation of PAT2 and two key studies using other amino acid transporters [34,35] identified tryptophan analogues as the most likely candidates. To allow identification of transported substrates and non-transported inhibitors, PAT2 was expressed in *Xenopus laevis* oocytes and transport function characterised using a combination of experimental approaches including radiotracer uptake and competition (cis-inhibition) studies, radiotracer efflux and trans-stimulation, and measurement of substrate-induced positive inward current using the two-electrode voltage-clamp (TEVC) technique.

## 2 Materials and methods

### 2.1 Materials

All amino acids, analogues and derivatives were purchased from Sigma-Aldrich (Poole, UK) except: guvacine and isoguvacine (Tocris, Bristol, UK); α-methyl-d,l-tryptophan, d- and l-pipecolic acids (Bachem, St. Helens, UK); and serotonin (Enzo Life Sciences, Exeter, UK). Radiolabelled amino acids used were: β-[3-^3^H]alanine (specific activity 50 Ci mmol^− 1^) and l-[2,3,4,5,6-^3^H]pipecolic acid (20 Ci mmol^− 1^) (American Radiolabeled Chemicals, St. Louis, USA); [2-^3^H]glycine (21 Ci mmol^− 1^) (GE Healthcare, Little Chalfont, UK); and l-[2,3,4,5-^3^H]proline (75–85 Ci mmol^− 1^) (PerkinElmer, Beaconsfield, UK).

### 2.2 Functional expression in *Xenopus laevis* oocytes

Female *Xenopus laevis* frogs were killed following Schedule 1 methods prior to surgical removal of the ovaries. Oocytes were prepared as described previously [1,27]. Individual stage V/VI oocytes were injected with 50 nl water (control) or cRNA (1 μg μl^− 1^) synthesised by in vitro transcription of pCRII containing mouse PAT1 [9] or pSPORT1 containing rat PAT2 [1] (there is 85% identity in amino acid sequence between human and mouse PAT1, and 81% identity between human and rat PAT2). Oocytes were maintained in modified Barth's solution (18 °C, 2–7 days) until use.

### 2.3 Radiolabel flux experiments

Oocytes were washed with Na^+^-free solution (in mM: 100 cholineCl; 2 KCl; 1 CaCl_2_; 1 MgCl_2_; 10 MES or 10 HEPES, adjusted to required pH with Tris base). Radiolabel uptake experiments were initiated by replacement of wash solution with 200 μl Na^+^-free solution (pH 5.5 unless stated otherwise) supplemented with [^3^H]amino acid (0.01–10 mM, 5 μCi ml^− 1^). For cis-inhibition experiments, uptake of [^3^H]amino acid was measured in the absence (control) and presence of competitor compound (0.01–30 mM). Unless stated otherwise, amino acids and derivatives are used routinely in their l-forms. Uptakes were measured over 40 min at 22 °C after which time the oocytes were washed three times with ice-cold Na^+^-free solution and lysed in 10% SDS before addition of scintillation cocktail (PerkinElmer). For trans-stimulation experiments, oocytes were pre-loaded with proline by microinjection of 50 nl l-[^3^H]proline (25 mM, 0.1 μCi μl^− 1^). Assuming an effective oocyte volume of 250 nl [36], this equates to an intracellular proline concentration of approximately 4 mM. Microinjected oocytes were allowed to recover (15 min) in 200 μl modified Barth's solution (18 °C). Individual oocytes were washed in Na^+^-free solution (pH 5.5) and incubated (22 °C, 10 min) in 100 μl Na^+^-free solution (pH 5.5) in the presence or absence of various compounds (all 10 mM). After the 10 min efflux period, the incubation solution was removed for scintillation counting and oocytes washed three times with ice-cold Na^+^-free solution. Oocytes were lysed in 10% SDS before addition of scintillation cocktail. Sample radioactivity was determined using an LS6500 liquid scintillation counter (Beckman Coulter, High Wycombe, UK).

### 2.4 Electrophysiology

Two-electrode voltage-clamp (TEVC) experiments were performed as described previously [25,27]. Water or cRNA-injected oocytes were continuously superfused with Na^+^-free solution (composition as per radiolabel flux experiments) whilst clamped at a membrane potential of − 60 mV. Current changes evoked by exposure (60–120 s) to experimental compounds (0.2–30 mM) were recorded using pClamp software connected to a Geneclamp 500 amplifier and Digidata 1200 (Axon Instruments, Molecular Devices, Sunnyvale, USA) and analysed using Clampfit 8.2 (Axon Instruments). Compound-induced current changes were calculated by subtraction of baseline (average current recorded over 15 s before addition of compound) from the average current recorded over the final 15 s of the exposure. Results are presented as cRNA-specific current (calculated by subtraction of current evoked in water-injected oocytes under identical experimental conditions) and representative trace recordings.

### 2.5 Data analyses

Data are mean ± SEM (n = number of oocytes), except where indicated in the text. Unless stated otherwise, results are expressed as the cRNA-specific response (following subtraction of response in water-injected oocytes under identical experimental conditions). Statistical significance was determined by unpaired or paired, two-tailed Student's t-test or one-way analysis of variance (ANOVA) with Tukey–Kramer multiple comparisons post-testing as appropriate. p < 0.05 was deemed statistically significant. Kinetic parameters were determined by fitting data to Michaelis–Menten kinetics. Statistical and kinetic analyses were performed using GraphPad Prism v.4 (GraphPad Software, San Diego, USA).

## 3 Results and discussion

### 3.1 Five-membered ring heterocyclic proline derivatives

**Fig. 1:**
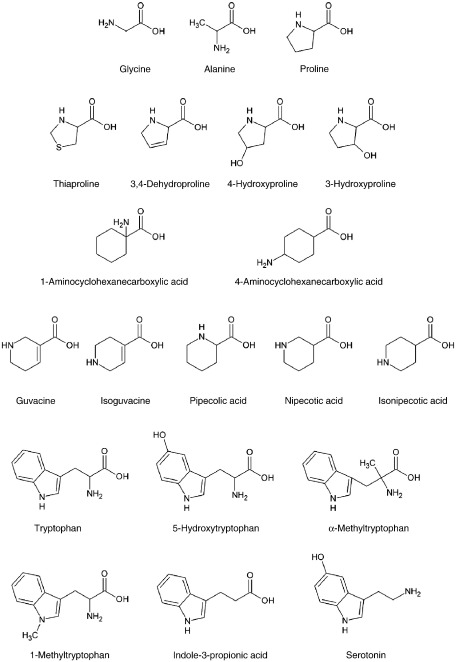
Structures of amino acids and derivatives (see PubChem website at http://pubchem.ncbi.nlm.nih.gov).

The substrate specificity of PAT2 has been characterised to some degree [1,9,26,27]. The major structural features that permit access and translocation of a compound by PAT2 include the presence of a free carboxyl head group, a small apolar side-chain, an amino head group in the α position, limited methylation of the amino group, and the distance between the charged carboxyl and amino head groups [1,9,26,27]. Of the twenty proteinogenic amino acids, glycine, l-alanine and l-proline are transported substrates for PAT2 with the five-membered ring heterocyclic l-proline (Fig. 1) having the highest affinity (K_m_ 0.1–0.2 mM) [1,9,27]. Generally, PAT2 has a preference for l-enantiomers of amino acids although this preference is weaker than that observed in most other mammalian amino acid carriers [9,26,27]. It is possible that the ring structure of proline might allow the substrate to be recognised in a different orientation within the PAT2 binding site or even in distinct orientations (as both an α- and a δ-imino acid) which might help explain why both d-proline and trans-4-hydroxy-l-proline have similar affinities for PAT2 to that observed with l-proline [26,27,37,38]. However, despite this high affinity for prolines, the maximum rate of l-proline transport via PAT2 is less than that observed with glycine [9,26,27]. This suggests that the heterocyclic structures (Fig. 1) are translocated less efficiently than glycine, perhaps due to some sterical hindrance within the transport pocket [26,27].

**Fig. 2:**
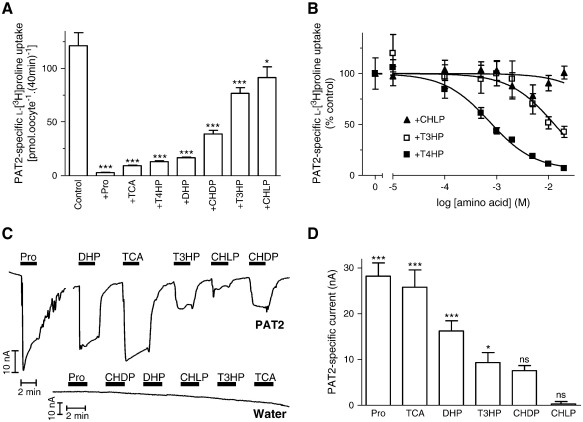
Interaction of proline derivatives with the amino acid transporter PAT2. A. l-[^3^H]Proline (100 μM) uptake (pH 5.5, Na^+^-free, 40 min) in PAT2-expressing oocytes in the absence (control) and presence of unlabelled proline (Pro) or related derivatives (all 10 mM), l-thiaproline (TCA), trans-4-hydroxy-l-proline (T4HP), 3,4-dehydro-d,l-proline (DHP), cis-4-hydroxy-d-proline (CHDP), trans-3-hydroxy-l-proline (T3HP) and cis-4-hydroxy-l-proline (CHLP). Results are expressed as PAT2-specific uptake (following subtraction of uptake in water-injected oocytes under identical experimental conditions). Data are mean ± SEM (n = 28–30). ***, p < 0.001; *, p < 0.05, versus control. B. Concentration-dependent inhibition of PAT2-mediated l-[^3^H]proline uptake (as in A above) by trans-4-hydroxy-l-proline (T4HP, filled squares, n = 20), trans-3-hydroxy-l-proline (T3HP, open squares, n = 7), and cis-4-hydroxy-l-proline (CHLP, filled triangles, n = 19) (all 0.01–20 mM). Results are expressed as a percentage of the uptake in the absence of inhibitor (after subtraction of uptake in water-injected oocytes). Data are mean ± SEM. C. Representative current traces from PAT2-expressing and water-injected oocytes upon exposure to proline and related derivatives (all 10 mM) measured by TEVC. D. Mean data for part C showing PAT2-mediated current (following subtraction of current induced in water-injected oocytes) evoked by proline and related derivatives. Data are mean ± SEM (n = 4–5). ***, p < 0.001; *, p < 0.05; ns, p > 0.05, versus current change in water-injected oocytes.

The lower turnover rate of PAT2 observed at high (mM) concentrations of heterocyclic prolines [9,26,27] suggests that heterocyclic amino acids are potential lead candidates in the identification of PAT2 inhibitors. l-[^3^H]Proline uptake (over 40 min) under optimal transport conditions (pH 5.5, Na^+^-free solution) via PAT2 was increased 17-fold above that in water-injected oocytes. PAT2-mediated l-[^3^H]proline uptake was reduced by the proline derivatives (all 10 mM) l-thiaproline (l-thiazolidine-4-carboxylic acid, TCA), trans-4-hydroxy-l-proline (T4HP), 3,4-dehydro-d,l-proline (DHP), cis-4-hydroxy-d-proline (CHDP), trans-3-hydroxy-l-proline (T3HP) and cis-4-hydroxy-l-proline (CHLP) (Fig. 2A). Modification within the ring structure (replacement of a carbon atom by sulphur in l-thiaproline; inclusion of the double-bond between the β- and γ-carbon atoms in 3,4-dehydro-d,l-proline; or oxidation of the γ-carbon in trans-4-hydroxy-l-proline) was well tolerated although the position and orientation of the hydroxyl group (Fig. 1) in the pyrrolidine ring (as observed in trans-4-hydroxy-l-proline, cis-4-hydroxy-d-proline, trans-3-hydroxy-l-proline and cis-4-hydroxy-l-proline) affected the relative affinity of the compounds for PAT2 (Fig. 2A–B). Trans-4-hydroxy-l-proline, trans-3-hydroxy-l-proline and cis-4-hydroxy-l-proline inhibited PAT2-mediated l-[^3^H]proline uptake with IC_50_ values of 0.7 ± 0.2, 13.1 ± 4.2 and > 20 mM, respectively. Competition experiments alone do not discriminate between cis-inhibition by a transported substrate or a non-transported inhibitor. To measure PAT2-mediated transport of these proline derivatives, substrate-induced inward currents were measured by TEVC where the net charge movement across the plasma-membrane is due to H^+^ influx coupled to amino acid import [9]. The relative inhibitory effect of each proline derivative was matched by its ability to evoke inward current in PAT2-expressing oocytes (Fig. 2C–D) (note that the current induced by trans-4-hydroxy-l-proline (T4HP) was demonstrated previously [27] to be equivalent to that generated by proline and is not shown here). No amino acid was able to induce current in water-injected oocytes (Fig. 2C). The close correlation between inhibitory potency and induction of current demonstrates that this group of compounds is unlikely to yield a non-transported inhibitor of PAT2.

### 3.2 Six-membered ring aminocyclohexanecarboxylic acids

**Fig. 3:**
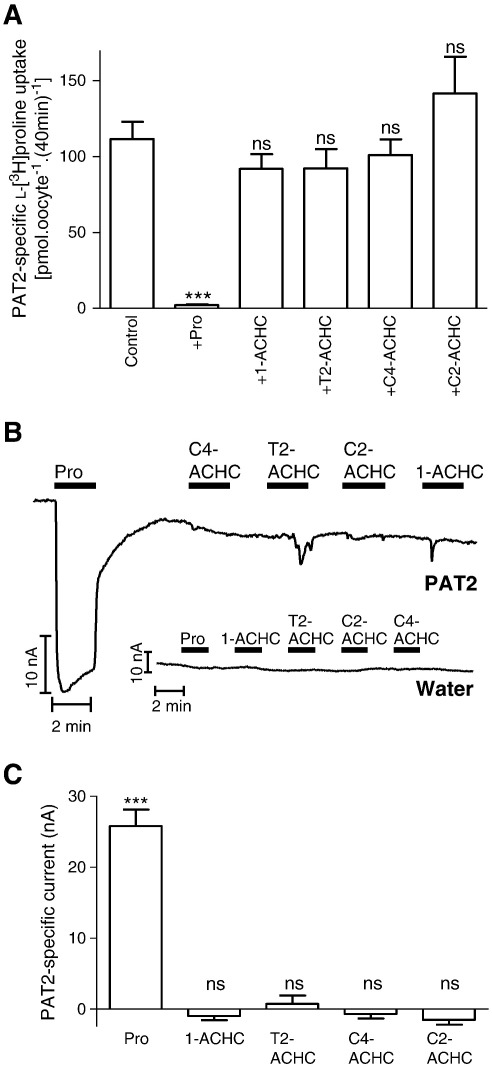
Aminocyclohexanecarboxylic acids (ACHCs) do not interact with PAT2. A. l-[^3^H]Proline (100 μM) uptake (pH 5.5, Na^+^-free) was measured in the absence (control) and presence of proline (Pro) and various ACHCs (all 10 mM), 1-aminocyclohexanecarboxylic acid (1-ACHC), trans-2-aminocyclohexanecarboxylic acid (T2-ACHC), cis-4-aminocyclohexanecarboxylic acid (C4-ACHC) and cis-2-aminocyclohexanecarboxylic acid (C2-ACHC). Data are mean ± SEM (n = 18–20). ***, p < 0.001; ns, p > 0.05, versus control. B. Representative current traces observed using PAT2 cRNA- or water-injected oocytes during exposure to proline (Pro) and various aminocyclohexanecarboxylic acids (all 10 mM). C. Mean data for part B showing PAT2-specific current change. Data are mean ± SEM (n = 4). ***, p < 0.001; ns, p > 0.05, versus current change in water-injected oocytes.

Previous observations [27] suggest that the four-membered ring heterocyclic compounds d-cycloserine and l-2-azetidine carboxylate are excellent PAT2 substrates. Thus, to try and identify non-transported inhibitors, we focussed on amino acid analogues containing bulkier ring structures. The six-membered ring cis-3-aminocyclohexanecarboxylic acid is used to selectively discriminate between GABA transport by the four GAT transporters (GAT1–4; SLC6A1, SLC6A13, SLC6A11 and SLC6A12, respectively). Cis-3-aminocyclohexanecarboxylic acid has a higher affinity for GAT1 whereas β-alanine has a preference for GAT3 and 4 [39]. We were unable to obtain cis-3-aminocyclohexanecarboxylic acid for this investigation but we examined a series of related aminocyclohexanecarboxylic acid (ACHC) compounds to determine what effect positioning the amino group outside the cyclic ring structure (Fig. 1) had on PAT2 (in the proline derivatives investigated in Fig. 2 the amino nitrogen is present within the heterocyclic ring). The ACHCs tested (1-aminocyclohexanecarboxylic acid, 1-ACHC; trans-2-aminocyclohexanecarboxylic acid, T2-ACHC; cis-4-aminocyclohexanecarboxylic acid, C4-ACHC; cis-2-aminocyclohexanecarboxylic acid, C2-ACHC) had no significant (p > 0.05) effect on PAT2-mediated l-[^3^H]proline uptake (Fig. 3A). In addition, all ACHCs investigated failed to induce inward current in PAT2-expressing oocytes (Fig. 3B–C). The reason for the lack of interaction of these compounds with PAT2 is not known but it is possible that the extra-cyclic nature of the amino nitrogen (Fig. 1) will allow the six-membered ring to be recognised as a bulky side-chain which, like bulky side-chain containing α-amino acids, is not tolerated by PAT2.

### 3.3 Six-membered heterocyclic GABA analogues

**Fig. 4:**
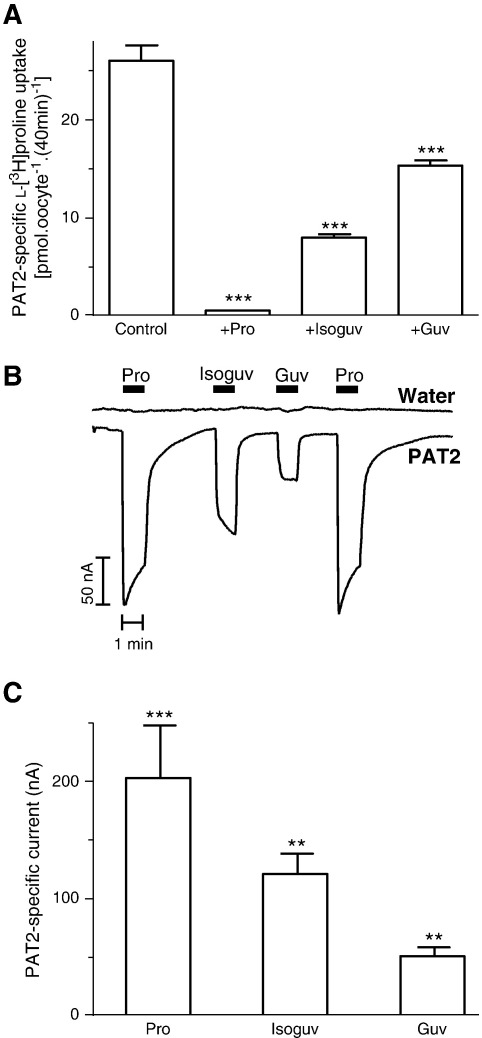
Interaction of guvacine and isoguvacine with PAT2. A. l-[^3^H]Proline (10 μM) uptake (pH 5.5, Na^+^-free) was measured in the absence (control) and presence of proline (Pro), isoguvacine (Isoguv) and guvacine (Guv) (all 10 mM). Data are mean ± SEM (n = 20). ***, p < 0.001, versus control. B. Representative current trace observed using PAT2-expressing and water-injected oocytes during exposure to proline (Pro), isoguvacine (Isoguv) and guvacine (Guv) (all 10 mM). C. Mean data for part B showing PAT2-specific currents. Data are mean ± SEM (n = 4). ***, p < 0.001; **, p < 0.01, versus water-injected oocytes.

Other heterocyclic GABA-related compounds are used to selectively inhibit GAT transporters (such as guvacine) or as agonists for GABA_A_ receptors (such as isoguvacine) [39–41]. In contrast to the ACHCs (Fig. 3), the amino nitrogen in guvacine and isoguvacine is contained within the heterocyclic ring (which also contains an unsaturated double-bond) (Fig. 1). Guvacine (Guv) and isoguvacine (Isoguv) (both 10 mM) significantly (p < 0.001) inhibited l-[^3^H]proline uptake (Fig. 4A) and the level of inhibition was consistent with the ability to induce inward current in PAT2-expressing oocytes (Fig. 4B–C) suggesting that both compounds are low affinity transported substrates of PAT2.

The six-membered heterocyclic ring (saturated) piperidine carboxylates pipecolic acid, nipecotic acid (a GAT transport inhibitor with a similar structure to guvacine) and isonipecotic acid (a GABA_A_ receptor agonist with a similar structure to isoguvacine) differ from each other by the distance between the amino and carboxyl groups (Fig. 1). Our previous investigation demonstrated that isonipecotic acid, nipecotic acid, and d- and l-pipecolic acids all inhibited PAT2-mediated l-[^3^H]proline uptake [27] with l-pipecolic acid being the most potent inhibitor (IC_50_ 1.2 mM). However, all four compounds were relatively poor at inducing inward currents via PAT2 [27]. This observation suggested that these heterocyclic compounds were only weakly translocated substrates for PAT2 and that they could prove useful lead compounds in the development of PAT2 inhibitors.

**Fig. 5:**
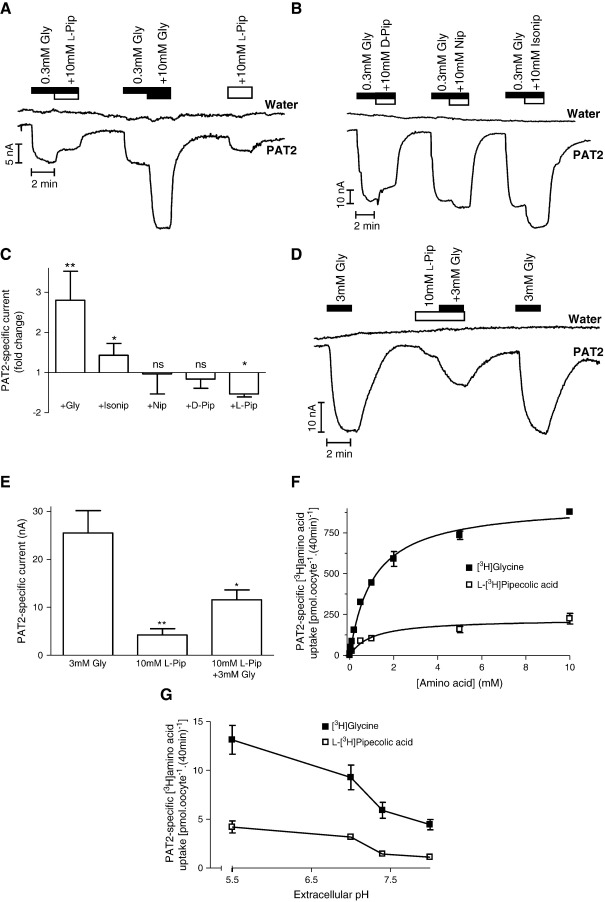
Interaction of piperidine carboxylates with PAT2. Representative current traces using PAT2-expressing and water-injected oocytes during exposure to glycine (Gly, 0.3 or 10 mM) and/or: A. l-pipecolic acid (l-Pip); B. d-pipecolic acid (d-Pip), nipecotic acid (Nip), isonipecotic acid (Isonip) (all 10 mM). C. Mean PAT2-specific current observed in parts A–B. Data represent the relative change in current observed when glycine (+ Gly), isonipecotic acid (+ Isonip), nipecotic acid (+ Nip), d-pipecolic acid (+ d-Pip) or l-pipecolic acid (+ l-Pip) (all 10 mM) are added to the superfusate in the continued presence of 0.3 mM glycine (control). Results are expressed as the fold change in current relative to that caused by 0.3 mM glycine. Data for glycine (n = 10) and l-pipecolic acid (n = 5) are mean ± SEM, and for isonipecotic acid, nipecotic acid and d-pipecolic acid are mean ± SD (n = 3). **, p < 0.01; *, p < 0.05; ns, p > 0.05, versus the current observed in the presence of 0.3 mM glycine (paired, two-tailed Student's t-test). D. Representative current traces using PAT2-expressing or water-injected oocytes during exposure to a saturating concentration (3 mM) of glycine (Gly) in the presence and absence of 10 mM l-pipecolic acid (l-Pip). E. Mean data for part D showing PAT2-specific current. Data are mean ± SEM (n = 4). **, p < 0.01; *, p < 0.05, versus the current observed in the presence of 3 mM glycine alone. F. [^3^H]Glycine (filled squares) and l-[^3^H]pipecolic acid (open squares) (both 0.01–10 mM) uptake (pH 5.5, Na^+^-free) in PAT2-expressing oocytes. Results are expressed as PAT2-specific uptake (following subtraction of uptake in water-injected oocytes). Data are mean ± SEM (n = 10). G. pH-Dependent [^3^H]glycine (filled squares) and l-[^3^H]pipecolic acid (open squares) (both 10 μM) uptake in PAT2-expressing oocytes at extracellular pH 5.5–8.0 (Na^+^-free). Results are expressed as PAT2-specific uptake. Data are mean ± SEM (n = 9–10).

When glycine in the superfusate is increased from a sub-saturating (0.3 mM) to a saturating (10 mM) concentration (K_m_ for glycine 0.5–0.7 mM) [1,9] the increase in glycine-induced current (p < 0.01) represents an increase from sub-maximal to maximal PAT2 transport (Fig. 5A and C). In contrast, when the total amino acid concentration is increased from the sub-saturating 0.3 mM concentration of glycine to the saturating concentration of l-pipecolic acid (l-Pip) (10 mM l-pipecolic acid plus 0.3 mM glycine) a decrease in the amino acid-induced current is observed (Fig. 5A and C). The current induced by 10 mM l-pipecolic acid is significantly smaller (p < 0.001) than that observed in the presence of 10 mM glycine (Fig. 5A). Similar observations were made with d-pipecolic (d-Pip), nipecotic (Nip) and isonipecotic (Isonip) acids (Fig. 5B–C). The apparent inhibitory effect (presumably due to a reduced rate of translocation) of l-pipecolic acid is confirmed in Fig. 5D–E where the current induced by 10 mM l-pipecolic acid alone is significantly less (p < 0.01) than that observed following exposure to 3 mM glycine. In addition, the current induced by the combination of saturating concentrations of l-pipecolic acid and glycine (10 and 3 mM, respectively) is significantly less (p < 0.05) than that induced by 3 mM glycine alone (Fig. 5D–E). In this particular group of compounds, the distance between polar head groups appears important as the greatest restriction to movement through the transporter occurs with l-pipecolic acid whereas elongation of the distance between carboxyl and amino groups, as observed in isonipecotic acid (Fig. 1), improves translocation (as measured by the isonipecotic acid-evoked current) (Fig. 5B–C).

It is possible that, rather than a slow translocation, the low level of current observed in the presence of l-pipecolic acid is due to a dissociation of amino acid and proton transport via PAT2 such that l-pipecolic acid is transported in a partially uncoupled mode. However, that does not appear to be the case as PAT2-mediated l-[^3^H]pipecolic acid uptake is much reduced (p < 0.001) compared to [^3^H]glycine due to a reduction in V_max_ from 934 ± 22 to 224 ± 19 pmol oocyte^− 1^ (40 min)^− 1^ (Fig. 5F). Thus, the levels of uptake of radiotracer forms of l-pipecolic acid and glycine are comparable to the relative levels of current induced by the two compounds demonstrating that there is no uncoupling of PAT2 transport. Finally, the low level of l-[^3^H]pipecolic acid uptake was not due to altered pH-dependence of the transporter as the uptake of l-[^3^H]pipecolic acid and [^3^H]glycine had an identical pattern over the pH range (5.5–8.0) investigated (Fig. 5G). The weakly transported nature of l-pipecolic acid via PAT2 demonstrates that it will have limited utility as a tool to provide unequivocal evidence for PAT2 function in native tissues. However, it should be useful when used in conjunction with other substrates and inhibitors and it is a good lead structure to aid the design of novel PAT2 inhibitors. The handling of l-pipecolic acid also distinguishes between PAT1 and PAT2 [14,27]. In PAT1, the levels of l-pipecolic acid translocation and competition are comparable, whereas in PAT2 it is a more effective blocker than transported substrate.

### 3.4 Tryptophan derivatives are non-transported inhibitors of PAT2

**Fig. 6:**
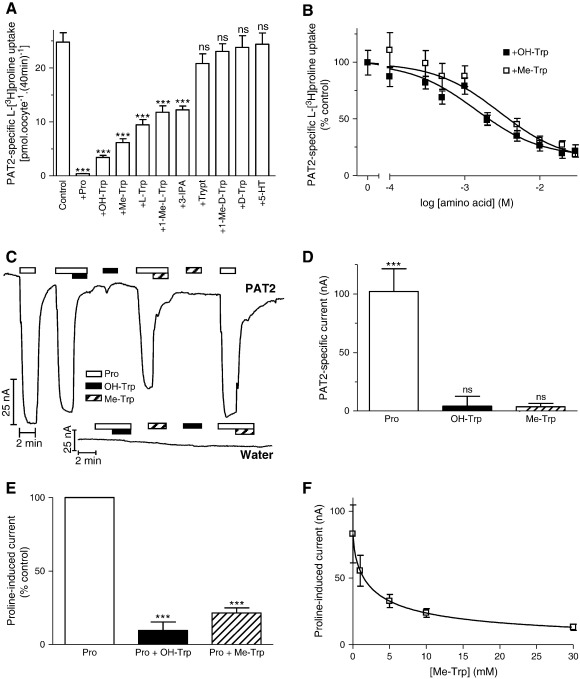
Interaction of tryptophan and related derivatives with PAT2. A. l-[^3^H]Proline (10 μM) uptake (pH 5.5, Na^+^-free) in PAT2-expressing oocytes in the absence (control) and presence of proline (Pro) or tryptophan and related derivatives (all 10 mM), 5-hydroxy-l-tryptophan (OH-Trp), α-methyl-d,l-tryptophan (Me-Trp), l-tryptophan (l-Trp), 1-methyl-l-tryptophan (1-Me-l-Trp), indole-3-propionic acid (3-IPA), tryptamine (Trypt), 1-methyl-d-tryptophan (1-Me-d-Trp), d-tryptophan (d-Trp) and serotonin (5-HT). Results are expressed as PAT2-specific uptake. Data are mean ± SEM (n = 19–20). ***, p < 0.001; ns, p > 0.05, versus control. B. l-[^3^H]Proline (10 μM) uptake (pH 5.5, Na^+^-free) in the absence and presence of 5-hydroxy-l-tryptophan (OH-Trp) or α-methyl-d,l-tryptophan (Me-Trp) (both 0.1–30 mM). Results are expressed as a percentage of the uptake in the absence of inhibitor (control). Data are mean ± SEM (n = 19–20). C. Representative current traces in PAT2-expressing or water-injected oocytes (pH 5.5, Na^+^-free) during exposure to 0.2 mM proline (Pro, open bar) in the presence or absence of 20 mM OH-Trp (solid bar) and 20 mM Me-Trp (hatched bar). D. Mean PAT2-specific current evoked by proline (0.2 mM, open column), OH-Trp (filled column) or Me-Trp (hatched column) (both 20 mM), as shown in part C. Data are mean ± SEM (n = 5–6). ***, p < 0.001; ns, p > 0.05, versus water-injected oocytes. E. Mean PAT2-mediated current (in part C) when oocytes are exposed to proline (0.2 mM) in the absence (Pro, open column) or presence of either OH-Trp (Pro + OH-Trp, filled column) or Me-Trp (Pro + Me-Trp, hatched column) (both 20 mM). Results are expressed as a percentage of the control (the current observed in the presence of 0.2 mM proline alone). ***, p < 0.001 versus control. Data are mean ± SEM (n = 5–6). F. Concentration-dependent inhibition by Me-Trp (1–30 mM) of PAT2-mediated proline-induced current (0.2 mM). Data are mean ± SEM (n = 5).

Recently, certain members of a separate group of heterocyclic amino acids, including the aromatic amino acid tryptophan and its derivatives (all of which contain an indole scaffold) (Fig. 1), have been identified as non-transported inhibitors of the amino acid transporters PAT1 (SLC36A1) and ATB^0,+^ (SLC6A14), respectively [34,35]. This group of compounds was investigated to determine their effects on PAT2 function. l-[^3^H]Proline uptake was inhibited significantly (p < 0.001) by 5-hydroxy-l-tryptophan (OH-Trp), α-methyl-d,l-tryptophan (Me-Trp), l-tryptophan (l-Trp), 1-methyl-l-tryptophan (1-Me-l-Trp) and indole-3-propionic acid (3-IPA) (all 10 mM) whereas the d-enantiomers d-tryptophan (d-Trp) and 1-methyl-d-tryptophan (1-Me-d-Trp) were without effect (p > 0.05) (Fig. 6A). The presence of the carboxyl group seems important in PAT2 binding as the decarboxylated amines tryptamine (Trypt) and serotonin (5-hydroxytryptamine, 5-HT) (both 10 mM) were without effect (p > 0.05) on l-[^3^H]proline uptake (Fig. 6A) whereas they interact with PAT1 with reasonable affinity [34]. Methylation of the α carbon atom (α-methyl-d,l-tryptophan) or nitrogen within the indole ring structure (1-methyl-l-tryptophan) (Fig. 1) was tolerated. The inhibition observed with indole-3-propionic acid suggests that the amino nitrogen on the α carbon atom is not a prerequisite for PAT2 binding.

The greatest level of inhibition of PAT2-mediated amino acid uptake was observed with the tryptophan derivatives 5-hydroxy-l-tryptophan and α-methyl-d,l-tryptophan and their interaction with PAT2 was investigated further. 5-Hydroxy-l-tryptophan and α-methyl-d,l-tryptophan inhibited l-[^3^H]proline uptake in a concentration-dependent manner with IC_50_ values of 1.6 ± 0.4 and 3.5 ± 1.5 mM, respectively (Fig. 6B). Neither 5-hydroxy-l-tryptophan nor α-methyl-d,l-tryptophan (both 20 mM) alone were able to elicit significant (p > 0.05 versus water-injected oocytes) current in PAT2-expressing oocytes (Fig. 6C–D). However, both 5-hydroxy-l-tryptophan and α-methyl-d,l-tryptophan (both 20 mM) caused significant (p < 0.001) reductions in current evoked by 0.2 mM l-proline (Fig. 6C and E). α-Methyl-d,l-tryptophan inhibited the l-proline-induced current with an IC_50_ of 2.7 ± 0.1 mM (Fig. 6F).

**Fig. 7:**
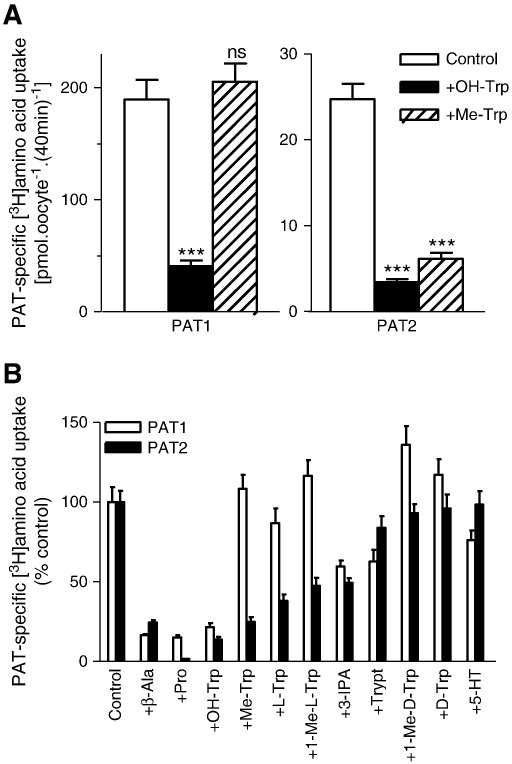
Inhibition of PAT1- and PAT2-mediated amino acid uptake by tryptophan derivatives. A. [^3^H]β-Alanine (100 μM, for PAT1) and l-[^3^H]proline (10 μM, for PAT2) uptake were measured (pH 5.5, Na^+^-free) in the absence (control, open columns) and presence of 5-hydroxy-l-tryptophan (OH-Trp, filled columns) or α-methyl-d,l-tryptophan (Me-Trp, hatched columns) (both 10 mM). Data are mean ± SEM (n = 20). ***, p < 0.001; ns, p > 0.05, versus control. B. PAT1 (open columns) and PAT2 (filled columns) mediated amino acid uptake (conditions as in A) in the absence (control) and presence of β-alanine (β-Ala), proline (Pro), 5-hydroxy-l-tryptophan (OH-Trp), α-methyl-d,l-tryptophan (Me-Trp), l-tryptophan (l-Trp), 1-methyl-l-tryptophan (1-Me-l-Trp), indole-3-propionic acid (3-IPA), tryptamine (Trypt), 1-methyl-d-tryptophan (1-Me-d-Trp), d-tryptophan (d-Trp) and serotonin (5-HT) (all 10 mM). Data are mean ± SEM (n = 19–20). Results are expressed as percent control (uptake in absence of inhibitor).

A comparison of the effects of 5-hydroxy-l-tryptophan and α-methyl-d,l-tryptophan on amino acid transport by PAT1 and PAT2 demonstrates that both tryptophan derivatives (10 mM) inhibit PAT2 with similar efficacy whereas 5-hydroxy-l-tryptophan causes a significantly greater (p < 0.001) inhibition of amino acid uptake via PAT1 than α-methyl-d,l-tryptophan (Fig. 7A). Extension of this comparison to include all of the tryptophan derivatives used in Fig. 6 allows the demonstration of a clear difference in the inhibition profile observed against amino acid uptake via PAT1 and PAT2 (Fig. 7B).

The ability of 5-hydroxy-l-tryptophan and α-methyl-d,l-tryptophan to inhibit PAT2-mediated amino acid transport and current, combined with the lack of inward current observed, suggests either that they represent non-transported blockers of PAT2 function or that they are PAT2 substrates transported in an electroneutral manner.

### 3.5 Discrimination between transported substrates and non-transported blockers of PAT2

**Fig. 8:**
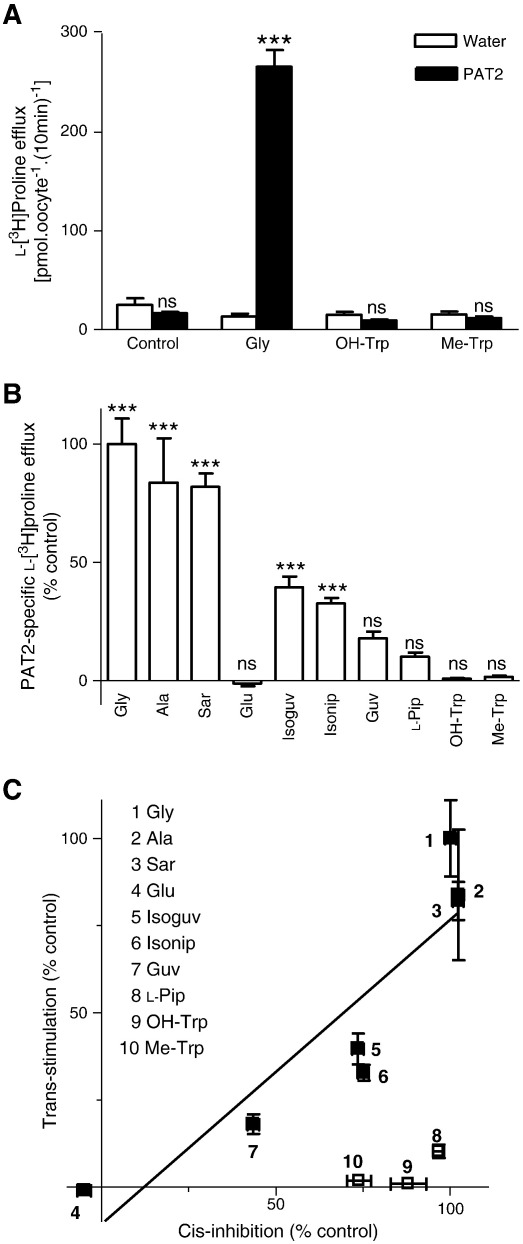
Trans-stimulation of PAT2-mediated l-[^3^H]proline efflux from oocytes discriminates between PAT2 substrates and non-transported inhibitors. PAT2-expressing and water-injected oocytes pre-loaded with l-[^3^H]proline (approximately 4 mM) were incubated (10 min) in pH 5.5 Na^+^-free solution in the presence or absence of various amino acids (all 10 mM). A. A typical trans-stimulation experiment to illustrate efflux of l-[^3^H]proline from PAT2-expressing (filled columns) and water-injected (open columns) oocytes in the absence (control) or presence of glycine (Gly), 5-hydroxy-l-tryptophan (OH-Trp) or α-methyl-d,l-tryptophan (Me-Trp) (all 10 mM). Data are mean ± SEM (n = 10). ***, p < 0.001; ns, p > 0.05, versus water-injected oocytes. B. l-[^3^H]Proline efflux was measured in the absence (control) or presence of glycine (Gly), l-alanine (Ala), sarcosine (Sar), glutamic acid (Glu), isoguvacine (Isoguv), isonipecotic acid (Isonip), guvacine (Guv), l-pipecolic acid (l-Pip), 5-hydroxy-l-tryptophan (OH-Trp) and α-methyl-d,l-tryptophan (Me-Trp) (all 10 mM). Results are expressed as the percentage of l-[^3^H]proline efflux in the presence of 10 mM extracellular glycine. Data are mean ± SEM (n = 5–10). ***, p < 0.001; ns, p > 0.05, versus efflux under control conditions (efflux from water-injected oocytes under identical conditions). C. Relationship between the abilities of various amino acids and derivatives to cis-inhibit PAT2-mediated amino acid uptake and trans-stimulate PAT2-mediated amino acid efflux. For cis-inhibition, l-[^3^H]proline (10 μM) uptake (pH 5.5, Na^+^-free) was measured in PAT2-expressing oocytes in the absence and presence of unlabelled amino acids (all 10 mM). Results were determined as PAT2-specific uptake (following subtraction of uptake in water-injected oocytes under identical experimental conditions) and are expressed as a percent of the inhibition observed in the presence of 10 mM glycine. Data are mean ± SEM (n = 20). For trans-stimulation, data are taken from panel B, and are expressed as the percent of the trans-stimulation observed in the presence of 10 mM glycine. The relationship [excluding l-pipecolic acid (l-Pip), 5-hydroxy-l-tryptophan (OH-Trp) and α-methyl-d,l-tryptophan (Me-Trp)] was analysed by linear regression (r^2^ = 0.82).

As suggested above, a potential limitation of measurement of substrate-induced currents by the TEVC technique is that electroneutral movement of compounds through the carrier cannot be detected. This has been demonstrated previously where H^+^-coupled short-chain fatty acid transport via both PAT1 and PAT2 occurs in an electroneutral fashion [42]. To address this issue, we determined the ability of various compounds to trans-stimulate l-[^3^H]proline efflux from oocytes pre-loaded by microinjection. In the absence of external amino acids, there was no significant (p > 0.05) difference in l-[^3^H]proline efflux from PAT2-expressing (22 ± 3 (n = 50) pmol oocyte^− 1^ (10 min)^− 1^) or water-injected (21 ± 2 (n = 50) pmol oocyte^− 1^ (10 min)^− 1^) oocytes (Fig. 8A). However, when the prototypical PAT2 substrate glycine (10 mM) was added to the extracellular compartment, a significant increase (p < 0.001) in l-[^3^H]proline efflux was observed from PAT2 (272 ± 10 (n = 50) pmol oocyte^− 1^ (10 min)^− 1^) but not water-injected (19 ± 2 (n = 50) pmol oocyte^− 1^ (10 min)^− 1^) oocytes, which represents a 14-fold increase in efflux. An example is shown in Fig. 8A where the presence of extracellular glycine (10 mM) is shown to increase l-[^3^H]proline efflux whereas 5-hydroxy-l-tryptophan and α-methyl-d,l-tryptophan (both 10 mM) have no effect (p > 0.05 versus water-injected oocytes). These data are consistent with the inhibitory effects of 5-hydroxy-l-tryptophan and α-methyl-d,l-tryptophan on amino acid-induced current via PAT2 (Section 3.4) being due to the two compounds acting as non-transported blockers of PAT2. Fig. 8B demonstrates that the ability of a compound in the extracellular compartment to stimulate l-[^3^H]proline efflux via PAT2 appears related to its ability to undergo PAT2-mediated translocation. Thus, the PAT2 substrates glycine (Gly), l-alanine (Ala) and sarcosine (Sar) all trans-stimulate l-[^3^H]proline efflux (Fig. 8B) whereas glutamic acid (Glu), which is excluded from PAT2, has no effect (Fig. 8B). The weakly transported heterocyclic compounds stimulate relatively low levels of efflux with isonipecotic acid (Isonip) being more effective than l-pipecolic acid (l-Pip). A direct relationship between the relative ability of compounds to cis-inhibit PAT2-mediated amino acid uptake and to trans-stimulate PAT2-mediated amino acid efflux can be observed in Fig. 8C. A deviation from the linear relationship, as observed with both 5-hydroxy-l-tryptophan and α-methyl-d,l-tryptophan, provides evidence for the inhibitory effects being due to the compounds acting as transport blockers rather than transported substrates.

## 4 Conclusions

A number of proline, GABA and tryptophan derivatives were examined to determine if they function either as transported substrates or non-transported inhibitors of the H^+^-coupled amino acid transporter PAT2. In general, the proline analogues were transported substrates with varying affinity for the transporter whereas the GABA analogues, in particular l-pipecolic acid, appear to be translocated relatively slowly. In contrast, both 5-hydroxy-l-tryptophan and α-methyl-d,l-tryptophan function as non-transported inhibitors of PAT2. Although the affinity of both compounds is relatively low, compared to the high affinity (IC_50_ approximately 250 μM) observed for the effect of α-methyl-d,l-tryptophan on amino acid transport via ATB^0,+^ [35], they are likely to prove highly useful experimental tools in the further investigation of the physiological roles of PAT2. Our knowledge of the physiological importance of PAT2 is limited although it seems likely to have roles in amino acid transport in both renal and neuronal tissues [26–28,31,32]. In addition, a recent study examining changes in gene expression in horses during implantation and early pregnancy demonstrates that the *SLC36A2* gene is highly upregulated in uterine tissues, perhaps reflecting the increased nutrient demands of the developing conceptus [43]. Understanding the substrate/inhibitor profile of a particular amino acid transporter has a number of potential uses in pathophysiological conditions as amino acid transporters can be utilised to mediate uptake of substrates that will alter cellular function or as a mechanism to deprive cells of essential nutrients [35]. Finally, the availability of non-transported inhibitors has utility in crystallographic studies, where the transport inhibitor can be used to trap a transporter protein in the open-to-out conformation, as observed with the leucine transporter LeuT [44].
